# Gene Expression Profiles of Intracellular and Membrane Progesterone Receptor Isoforms in the Mediobasal Hypothalamus During Pro-Oestrus

**DOI:** 10.1111/j.1365-2826.2009.01920.x

**Published:** 2009-12

**Authors:** B Liu, L A Arbogast

**Affiliations:** Department of Physiology, Southern Illinois University School of MedicineCarbondale, IL, USA

**Keywords:** oestradiol, oestrous cycle, arcuate nucleus, dopamine, prolactin

## Abstract

Progesterone action is mediated by its binding to specific receptors. Two progesterone receptor (PR) isoforms (PRA and PRB), three membrane progesterone receptor (mPR) subtypes (mPRα, mPRβ and mPRγ) and at least one progesterone membrane-binding protein [PR membrane component 1 (PRmc1)] have been identified in reproductive tissues and brain of various species. In the present study, we examined gene expression patterns for PR isoforms, mPR subtypes and PRmc1 in the rat mediobasal hypothalamus (MBH) during pro-oestrus. The mRNA level for each receptor subtype was quantified by a real-time reverse transcriptase-polymerase chain reaction (RT-PCR) at the time points: 13.00 h on dioestrous day 2; 09.00, 13.00, 17.00 and 22.00 h on pro-oestrus; and 13.00 h on oestrus. For PR, one primer set amplified PRA+PRB, whereas a second primer set amplified PRB. As expected, PRA+PRB mRNA expression was greater than PRB in MBH tissue. PRB mRNA levels increased throughout the day on pro-oestrus, with the highest levels being observed at 17.00 h. PRB mRNA levels in the MBH were increased by 2.4- and 3.0-fold at 13.00 and 17.00 h, respectively, on pro-oestrus compared to 13.00 h on dioestrous day 2. There were differential mRNA expression levels for mPRs and PRmc1 in the MBH, with the highest expression for PRmc1 and the lowest for mPRγ. The mPRα mRNA contents at 13.00 and 17.00 h on pro-oestrus were increased by 1.5-fold compared to that at 13.00 h on dioestrous day 2. The mPRβ mRNA levels at 13.00 and 17.00 h on pro-oestrus were 2.5- and 2.4-fold higher compared to that at 13.00 h on dioestrous day 2, respectively. PRA+PRB, mPRγ and PRmc1 mRNA levels did not vary on pro-oestrus. These findings suggest that the higher expression of PRB, mPRα and mPRβ in the MBH on pro-oestrous afternoon may influence both genomic and nongenomic mechanisms of progesterone action during the critical pre-ovulatory period.

Progesterone is involved in biological functions in the uterus, ovary, mammary gland and brain of various species (1). Many progesterone effects are mediated by the classical intracellular progesterone receptor (PR) isoforms. However, the recent identification of membrane progesterone receptors (mPR) provides another mechanism for progesterone action. PR-expressing cells in the brain are found in discrete brain regions, including the preoptic area, periventricular nucleus, anterior hypothalamus, ventromedial nucleus, arcuate nucleus and median eminence (2). The mediobasal hypothalamus (MBH) contains important populations of PR-containing neuroendocrine neurones implicated in luteinising hormone (LH) and prolactin release (3–7). The pre-ovulatory progesterone rise contributes to the amplification or extension of the LH and prolactin surges on pro-oestrus in rats (8–11)

Two PR isoforms are derived from a single gene and generated from alternative transcriptional and translational start sites (12–15). The structural difference between the PRA and PRB isoforms is an additional 164 amino acids located at the *N*-terminus of PRB. The differential expression of PRA and PRB among target tissues (16, 17) contributes to the appropriate cell-specific responses and distinct physiological functions (18–20). Oestradiol and progesterone have the capacity to regulate PR gene expression (2, 21–23). During the oestrous cycle, PRB was found to be the predominant PR isoform in the hypothalamus, preoptic area and frontal cortex and differential PRA and PRB expression were observed in the hypothalamus, preoptic area and the frontal cortex (17).

The rapid nongenomic progesterone actions initiated at the cell surface by binding to the membrane receptors have received increased attention in the past decade (24–26). The first mPR, designated mPRα, was cloned from spotted seatrout ovary and its protein met the designation as a steroid membrane receptor (27). Subsequently, mPR subtypes, mPRα, mPRβ and mPRγ, were identified in tissues including ovary, hypothalamus and pituitary gland (28–30). The mPR protein has an approximately 40 kDa molecular mass and seven transmembrane domains (28–30). In addition to the mPRs, a small progesterone-binding protein (25 kDa) may also mediate progesterone action (31). This progesterone-binding protein was initially identified from porcine vascular smooth muscle cells and liver microsomes (32, 33). This protein was referred to as PR membrane component 1 (PRmc1) and has one single transmembrane domain (34). PRmc1 was detected in the reproductive system (35, 36) and basal forebrain (37).

The present study explored PRs, mPRs and PRmc1 gene expression patterns in the MBH during oestrous cycle, especially on pro-oestrus. The study provides important information for understanding progesterone regulation of hypothalamic neuronal activities during the critical pre-ovulatory phase of the reproductive cycle.

## Materials and methods

### Animals

Adult female Sprague–Dawley rats (200–250 g) were obtained from Charles River (Raleigh, NC, USA). Rats were housed under a 14 : 10 h light/dark cycle (lights on 07.00 h) and controlled temperature. Food and water were supplied *ad lib*. The stage of the oestrous cycle was monitored by performing a daily vaginal lavage at 10.00–12.00 h. Only rats exhibiting three consecutive 4-day oestrous cycles were used in the study. Rats were killed by decapitation at 13.00 h on dioestrus 2, 09.00, 13.00, 17.00 and 22.00 h on pro-oestrus, or 13.00 h on oestrus. The brain was removed rapidly and frozen in Fisherbrand Super Friendly Freeze It solution (Fisher Scientific, Pittsburgh, PA, USA) and stored at −80 °C. MBH tissue was punched with a 2-mm diameter sample corer (Fine Science Tools, Foster City, CA, USA) from frozen brain equilibrated to −20 °C. The coordinates were −1.7 and −3.7 mm from Bregma and 1.0 mm laterally as determined from optic chiasm and lateral hypothalamic sulci on the ventral surface of the brain (38). The MBH core sample was placed under a dissecting microscope and cut at 1.5 mm from the ventral surface of the brain. The MBH fragment included the arcuate nucleus, periventricular nucleus, part of ventromedial hypothalamus, as well as the median eminence. Dissected MBH tissue was used for RNA isolation. All animal experiments were conducted according to the National Institutes of Health Guide for the Care and Use of Laboratory Animals. The Institutional Animal Care and Use Committee at Southern Illinois University at Carbondale approved the experimental protocols.

### RNA isolation

Total RNA was isolated from the MBH tissue using RNA Bee method (Tel-Test Inc., Friendswood, TX, USA) according to the manufacturer’s instructions. Briefly, MBH tissue was homogenised in samples in 0.8 ml RNA Bee solution using a glass homogeniser. Chloroform (80 μl) was added for phase separation and samples were centrifuged at 12 000 ***g*** (4 °C) for 15 min. RNA in the aqueous phase was precipitated with 0.4 ml isopropanol. After centrifugation, RNA precipitates were washed with 0.8 ml of 75% ethanol and RNA pellet was solubilised in water. RNA quantity was determined spectrophotometrically. RNA samples were stored at −80 °C until further use.

### cDNA synthesis

Complementary DNA (cDNA) was synthesised using SuperScript™ First-strand synthesis system (Invitrogen, Carlsbad, CA, USA). A 12-μl reaction, including 5 μg of RNA, 1 μl of oligo(dT)_12–18_ (25 μg/μl) and diethylpyrocarbonate-treated water was incubated at 70 °C for 10 min, followed by at least 1 min of incubation on ice. The cDNA synthesis mix, including 2 μl of (10×) reverse transcriptase buffer, 2 μl of 25 mm magnesium chloride, 1 μl of 10 mm deoxynucleotide triphosphates and 2 μl of 0.1 mm dithiothreitol, was added to the above reaction tube. The reaction mixture was then incubated at 42 °C for 5 min. After addition of 1 μl (200 units) SuperScript™ III reverse transcriptase, the reaction mixture was incubated at 42 °C for 50 min. The reaction was terminated by incubation at 70 °C for 15 min. RNAase H (1 μl) was added to each tube and the reaction mixture was incubated at 37 °C for 20 min. The cDNA samples were stored at −20 °C.

### Real-time polymerase chain (PCR) for PRs, mPRs and PRmc1

Real-time PCR was performed in the SmartCycler System (Cepheid, Sunnyvale, CA, USA) by utilising the SYBR® Green JumpStart™ Taq ReadyMix™ Kit (S 4438; Sigma, St Louis, MO, USA). The 25-μl reactions contained 12.5 μl of SYBR Green JumpStart Taq ReadyMix, 0.5 μl of each primer (0.2 μm final concentration), 1 μl of cDNA template and 10.5 μl of water. Primer sequences were designed using MacVector 9.5 software (MacVector Inc., Cary, NC, USA) and are presented in Table 1. After the initial denaturation step at 95 °C for 2 min, amplification was performed for 40 cycles of denaturation at 95 °C for 15 s, annealing at 60 °C for 30 s, and elongation at 72 °C for 30 s. The specificity and purity of the amplification reaction was determined by performing a melting curve analysis at 60 °C up to 95 °C at 0.5 °C increments. One distinct peak was observed, indicating that a single DNA sequence was amplified during PCR. In addition, end reaction products were visualised on ethidium bromide-stained 2% agarose gels and the appearance of a sole band of the correct molecular size was confirmed. Each PCR assay included two negative controls, without reverse transcription or without template in the PCR reaction. The data were analysed with SmartCycler Software. The comparative threshold cycle number (Ct) method (2^−ΔΔCt^) was used to quantify the results obtained by real-time RT-PCR (39). The gene expression patterns of PRA+PRB, PRB, mPRα, mPRβ, mPRγ and PRmc1 were normalised to that of glyceraldehyde 3-phosphate dehydrogenase (GAPDH). GAPDH mRNA levels did not change during the oestrous cycle in the MBH as determined from the mean Ct value for each group. The ΔCt in each sample was calculated by subtracting Ct^GAPDH^ from Ct^PRs, mPRs or PRmc1^. The gene expression changes were determined by subtracting ΔCt experimental groups (pro-oestrus and oestrus) from the ΔCT control group (13.00 h dioestrus 2). Fold induction was determined by calculating 2^−ΔΔCt^. The real-time PCR efficiency for each primer pair was calculated using the equation E = 10^−[1/s]^ (40), where ‘s’ is the slope derived from the SmartCycler rest-mcs software.

**Table 1:** Primer Sequences for Quantitative Real-time Polymerase Chain Reaction.

Gene name	GenBank accession no.	Primer sequence	Amplicon size (bp)
PRA+PRB	NM_022847	Forward: GGT CTA AGT CTC TGC CAG GTT TCC	182
		Reverse: CAA CTC CTT CAT CCT CTG CTC ATT C	
PRB	NM_022847	Forward: GCA TCG TCT GTA GTC TCG CCA ATA C	176
		Reverse: GCT CTG GGA TTT CTG CTT CTT CG	
PRα	NM_001034081	Forward: CAC ACT GTT CCA GCA GCA CAA C	143
		Reverse: CAA TGA TGA AGA GGG GCA GAG C	
PRβ	NM_001014099	Forward: CTC TTT CAG AAG CAC AAC GAG GTG	168
		Reverse: GGT GAG GTA AGT GAT TGA CGA CAG G	
PRγ	NM_001014092	Forward: CTC CTG GAC GCT TTG ACT ACA TTG	198
		Reverse: TGC TGA GGC TGA AGA TGA TGC	
PRmc1	NM_021766	Forward: ACT TCA CCC CTG CCG AAC TAA G	199
		Reverse: TCA TCC TTC AGT GCT TCT TTG TCC	
GAPDH	DQ403053	Forward: AAC GAC CCC TTC ATT GAC C	191
		Reverse: TCC ACG ACA TAC TCA GCA C	

### Statistical analysis

Data are presented as the mean ± SEM. Statistical significance was evaluated by one-way anova and multiple comparisons were made with Fisher’s least significant procedures. P < 0.05 was considered statistically significant.

## Results

### Standard curves for PRs, mPRs, PRmc1 and GAPDH cDNA

**Fig. 1:**
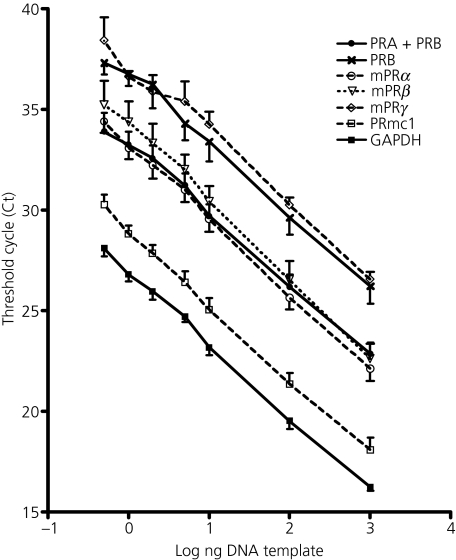
Standard curves for real-time polymerase chain reaction amplification of progesterone receptors (PRs), membrane progesterone receptors (mPRs) and PR membrane component 1 (PRmc1) in the mediobasal hypothalamus (MBH) tissue of rats. The lower Ct value represents higher mRNA concentrations of certain target genes in the tissue. As indicated on the figure, glyceraldehyde 3-phosphate dehydrogenase has the highest mRNA level in the MBH, followed in expression level order by PRmc1, PRα, PRA+PRB, PRβ, PRB, PRγ.

To establish standard curves for PRs, mPRs, PRmc1 and GAPDH gene expression in the MBH, we tested cDNA concentrations of 0.5, 1, 2, 5, 10, 100 and 1000 ng from mixed MBH cDNA samples, which included MBH tissues from dioestrus 2, pro-oestrus and oestrus. This study was repeated three times with different mixed cDNA samples. As shown in Fig. 1, a concentration-dependent change of Ct value was observed for PRA+PRB, PRB, mPRα, mPRβ, mPRγ, PRmc1 and GAPDH in MBH cDNA samples. The real-time PCR efficiencies of the primer sets for GAPDH, PRA+PRB, PRB, mPRα, mPRβ, mPRγ and PRmc1 were 1.89, 1.95, 1.93, 1.85, 1.81, 1.94 and 1.87, respectively. GAPDH, the housekeeping gene, and PRmc1 mRNA levels in the MBH were expressed at the highest levels compared to PRs and mPRs mRNA levels. As expected, because PRA+PRB primer set recognises a cDNA sequence common to both PRA and PRB, the expression of PRA+PRB was higher than PRB alone. The mRNA expression levels for mPRα and mPRβ were higher in MBH than mPRγ mRNA levels (Fig. 1).

### Expression of PR genes in the MBH

**Fig. 2:**
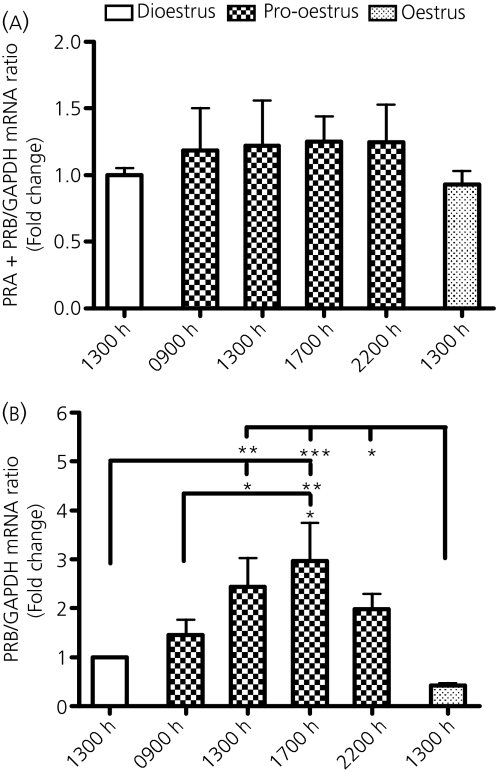
PRA+PRB (a) and PRB (b) gene expression patterns in the mediobasal hypothalamus of cycling female rats. Rats were killed at 13.00 h on dioestrus 2; 09.00, 13.00, 17.00 and 22.00 h on pro-oestrus; or 13.00 h on oestrus. Each value represents the mean ± SEM of determinations from seven to ten rats. *P < 0.05, **P < 0.01, ***P < 0.001 compared to the corresponding group indicated by a long vertical line.

Gene expression patterns for PRA+PRB and PRB in the MBH were evaluated at 13.00 h on dioestrus 2; 09.00 13.00, 17.00 and 22.00 h on pro-oestrus; and 13.00 h on oestrus. PRA+PRB mRNA contents in the MBH were similar on dioestrus 2, pro-oestrus and oestrus (Fig. 2a). By contrast, PRB mRNA contents were increased 1.5-, 2.4- and 3.0-fold at 09.00, 13.00 and 17.00 h, respectively, on pro-oestrus, compared to 13.00 h on dioestrus 2. PRB mRNA content at 22.00 h on pro-oestrus declined to 2.0-fold dioestrous levels, and was further reduced by 13.00 h on oestrus to 43% of dioestrous levels (F_5,39_ = 4.202, P < 0.01; Fig. 2b).

### Expression of mPR genes in the MBH

**Fig. 3:**
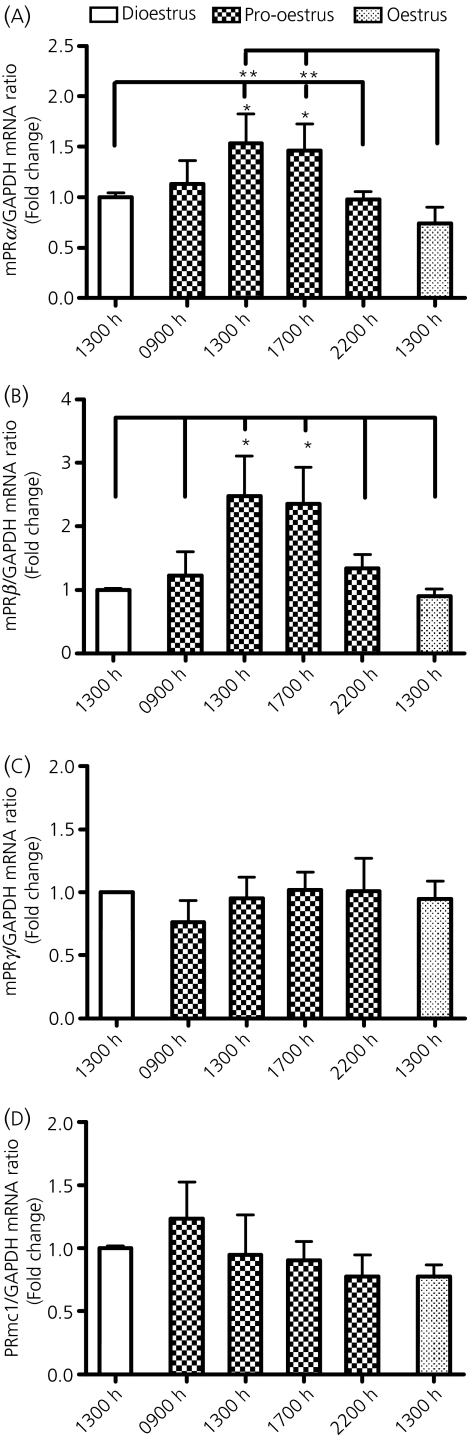
Gene expression patterns for mPRα (a), mPRβ (b), mPRγ (c) and PRmc1 (d) in the mediobasal hypothalamus of cycling female rats. Rats were killed at 13.00 h on dioestrus 2; 09.00, 13.00, 17.00 and 22.00 h on pro-oestrus; or 13.00 h on oestrus. Each values represents the mean ± SEM of determinations from seven to ten rats. *P < 0.05, **P < 0.01 compared to the corresponding group indicated by a long vertical line.

Gene expression of mPRs, including mPRα, mPRβ, mPRγ and PRmc1, in the MBH were also determined at 13.00 h on dioestrus 2; 09.00, 13.00, 17.00 and 22.00 h on pro-oestrus; and 13.00 h on oestrus. Analysis using one-way anova revealed significant changes in mRNA contents for mPRα (F_5,38_ = 2.494, P < 0.05; Fig. 3a) and mPRβ (F_5,39_ = 2.884, P < 0.05; Fig. 3b) in the MBH, but no changes were found for mPRγ (Fig. 3c) and PRmc1 (Fig. 3d) expression. Specifically, mPRα mRNA contents at 13.00 and 17.00 h on pro-oestrus were significantly higher than that at 13.00 h on dioestrus 2, 22.00 h on pro-oestrus and 13.00 h on oestrus, with a 1.5-fold increase at 13.00 and 17.00 h on pro-oestrus compared to that at 13.00 h on dioestrus 2. The mRNA contents for mPRβ at 13.00 and 17.00 h on pro-oestrus were higher than the values at all the other time points during the oestrous cycle. The mPRβ mRNA contents at 13.00 and 17.00 h were 2.5- and 2.4-fold higher than that at 13.00 h on dioestrus 2, respectively. PRmc1 and mPRγ mRNA levels were not altered on pro-oestrus.

## Discussion

The present study characterised gene expression patterns of PR isoforms (PRA+PRB and PRB), three mPR subtypes (mPRα, mPRβ and mPRγ) and PRmc1 in the rat MBH during the oestrous cycle, especially on pro-oestrus. The data obtained indicate that PRA, PRB, mPRα, mPRβ, mPRγ and PRmc1 are expressed in MBH tissue, albeit at different levels. PRB, mPRα and mPRβ expression in the MBH varied during the oestrous cycle with higher mRNA contents on pro-oestrous afternoon compared to that on pro-oestrous morning, early afternoon of dioestrus 2 and early afternoon of oestrus. This basic profile for PRs gene expression in the MBH during the reproductive cycle may be beneficial for understanding the mechanism(s) of steroid hormone regulating neuronal activity, hormone secretion and reproductive behaviours.

Of the classical intracellular PRs, PRB mRNA contents in the MBH fluctuated during the oestrous cycle, especially on pro-oestrus. The highest level of PRB mRNA expression occurred during pro-oestrous afternoon, but PRB mRNA contents declined on pro-oestrous evening and were markedly reduced by oestrous afternoon. These data are in general agreement with the previous study by Guerra-Araiza *et al.* (17) with respect to differential mRNA expression of PR isoforms during the oestrous cycle. These investigators examined PRB expression at 12.00 h on each day of the oestrous cycle and reported highest PRB mRNA levels in the hypothalamus on pro-oestrus compared to other days of the oestrous cycle. Our data expand these data to indicate that PRB expression continues to increase during pro-oestrous afternoon and reaches the highest levels coincident with the timing of LH and prolactin surges. It is not clear how these changes in PRB mRNA translate to functional PRB protein on pro-oestrous afternoon. Guerra-Araiza *et al.* (41) reported no change in PRB isoforms protein content in the hypothalamus at 12.00 h on pro-oestrus compared to 12.00 h on other days of the oestrous cycle. However, future studies should examine protein expression for PR and mPR isoforms throughout pro-oestrous day because elevated levels in the later afternoon might provide maximum sensitivity at the time of the pre-ovulatory progesterone rise. Early studies indicated that PR binding in the hypothalamus is highest on pro-oestrus and lowest on dioestrus (42). Because the entire PRA sequence is contained within the PRB sequence, primers were designed to amplify PRA+PRB. It is noteworthy that PRA+PRB mRNA contents were not significantly altered during pro-oestrous day or on different days of the oestrous cycle. These data suggest that PRA isoforms expression did not increase in a similar manner to PRB on pro-oestrus or potentially offset PRB expression changes. In either case, the data obtained in the present study suggest that the ratio of PRA : PRB might be altered during pro-oestrus and allow for distinct progesterone-dependent transcriptional changes in the MBH during the pre-ovulatory period.

The data obtained in the present study indicate that PRB may be more sensitive to endogenous steroid hormone variance. A number of studies reported that PR expression in the hypothalamus is dependent on prior oestradiol priming (2, 21, 22, 43). Oestradiol treatment of ovariectomised rats results in increased PR expression (22, 43, 44) and a high co-expression of PR with tyrosine hydroxylase (TH) in the arcuate nucleus (3, 4, 45). Oestrogen receptor-α primarily mediates oestradiol-mediated PR expression in specific hypothalamic nuclei, but other oestradiol receptor(s) or splice variant(s) may also contribute in some nuclei (46, 47). The action of oestradiol with respect to promoting PR expression was observed at 24 h post-injection in the arcuate and ventromedial nuclei of rat hypothalamus (22). In addition, a higher PRB mRNA content, as compared to PRA+PRB mRNA contents, was obtained in the hypothalamus/preoptic area within 12 h after oestradiol treatment (43). These data taken together with our data suggest that increased PRB expression on pro-oestrus afternoon may be a result of the high serum oestradiol that rises from late on dioestrus 2 (48) and is maintained until 19.00 h on pro-oestrus (49). Indeed, oestradiol enhances PRB gene expression in the preoptic area and hypothalamus (23, 43, 50). Two functionally distinct promoters have been identified in the rat PR gene with a degree of promoter specificity for oestrogen responsiveness (12, 14). These characteristics of the rat PR promoter may account for the ability of oestradiol to preferentially induce PRB expression in the MBH. By contrast to the action of oestrogen, progesterone inhibits PR gene expression in the hypothalamus (23). The pre-ovulatory progesterone rise that occurs from 17.00 h through 22.00 h (49) may contribute to the reduction of PRB mRNA levels in the MBH at 22.00 h on pro-oestrus. At 3 h after treatment, progesterone diminishes the protein content of PR isoforms in the hypothalamus of oestradiol-primed ovariectomised rats (41).

This is the first study to describe the gene expression patterns of mPRs and PRmc1 in the MBH. Our data show that mPRα and mPRβ gene expressions varied on pro-oestrous afternoon. The mPRα and mPRβ mRNA levels were higher at 13.00 and 17.00 h on pro-oestrus than at other time points tested during the oestrous cycle. Interestingly, the pro-oestrous changes in mPRα and mPRβ expression levels were similar to that of PRB and the profile of the changes is consistent with up-regulation by oestradiol and subsequent down-regulation by progesterone. Indeed, oestradiol increases mPRα and mPRβ mRNA expression in human myometrium, albeit with dissimilar timing and magnitude, whereas progesterone differentially modulates expression of these receptors (51). The identification and characterisation of mPRs is a recent development in our understanding of rapid progesterone signalling. The mPRs have characteristics of seven transmembrane cell surface receptors. Recently, mPR was demonstrated to directly couple to G_i_ protein to down-regulate membrane-bound adenylyl cyclase activity (52). Moreover, mPRα and mPRβ protein are found in the plasma membrane fraction of GT1-7 cells (53). Activity changes of protein kinase C and calcium and calmodulin-dependent protein kinase II in the ventromedial nucleus and preoptic area of the rat hypothalamus can be initiated by progesterone (54, 55). These studies imply that mPR-regulated intracellular signalling pathways may be sites for progesterone action in the MBH. Further studies are required before we fully understand the subcellular localisation, importance and regulation mPRα and mPRβ in the MBH during the reproductive cycle. Although there was a high expression of PRmc1 mRNA and a relatively low level of mPRγ mRNA in the MBH, their mRNA levels were not altered at the times examined during the rat oestrous cycle. However, PRmc1 expression in the ventromedial nucleus of the rat hypothalamus is up-regulated by oestradiol and down-regulated by progesterone (37). The MBH fragment in the present study included the arcuate nucleus as well as the ventromedial nucleus and the modest, albeit nonsignificant, changes in PRmc1 during pro-oestrus in the present study may represent a contribution from the ventromedial nucleus.

The data obtained in the present study indicate progressively increasing expression levels for PRB, mPRα and mPRβ throughout pro-oestrous afternoon. Peak expression occurs at the time of the onset of the pre-ovulatory rise in circulating progesterone levels and suggests that increased progesterone responsiveness might be bestowed to cells in the MBH by this subset of progesterone receptors. It is notable that oestradiol also increases 3β-hydroxy-steroid hydrogenase/Δ5-Δ4 isomerase in the hypothalamus/preoptic area of ovariectomised-adrenaleised rats and stimulates *de novo* progesterone synthesis in hypothalamic astrocyte cultures (43, 56, 57). The ovarian steroid hormones, oestradiol and progesterone, are essential for the expression and amplification/extension of LH and prolactin surges on pro-oestrus. The intracellular PRs are critically involved in LH surge in rats, which is blocked or attenuated by the intracellular PR antagonist, RU486 (50, 51). Blockade of local progesterone synthesis in the brain also results in decreased LH surge (57). The LH surge is absent in PR knockout mice on pro-oestrous day and in ovariectomised PR-knockout mice treated with steroid hormones (58). Recently, mPRs have been implicated in the PRA/PRB-independent rapid negative-feedback mechanisms responsible for the suppression of GnRH release (53). Given the timing of increases in mPRα and mPRβ expression just prior to the onset of the LH surge, our data point to the importance of examining the role of the mPRs in positive feedback-mechanisms responsible for LH secretion on pro-oestrus in future studies.

Our laboratory has an interest in understanding the regulation of the pre-ovulatory prolactin surge. Although the prolactin surge is absolutely dependent on oestradiol (48), progesterone is responsible for amplifying the magnitude or extending the duration of the surge by decreasing TH activity and TH phosphorylation state in tuberoinfundibular dopamine (TIDA) neurones of MBH (8, 9, 49). The progesterone-induced decrease in TIDA neuronal activity can be blocked by antisense oligonucleotides to progesterone receptor (59), suggesting that progesterone is acting via specific PR. The high co-expression of PR with TH in the MBH supports that the neuroendocrine dopaminergic neurones in the arcuate nucleus are the targets of progesterone action (3–5, 45, 60, 61). Noteworthy, the MBH tissue dissected in the present study included arcuate nucleus and median eminence, where dopaminergic neuronal body or terminals are located. Additionally, the colocalisation of PR neuropeptide Y (5), neurotensin (6) and dynorphin (62) in the arcuate nucleus indicates the potential importance of this study in a wide range of physiological functions.

Differential expression patterns for PRs and mPRs during oestrous cycle, especially on pro-oestrus, provide an important profile for understanding progesterone-mediated activity in the MBH. Whether these changes in PR and mPR mRNA levels translate into differences in functional protein levels for PR and mPR isoforms in the MBH remains to be explored in future studies. The present study suggests that increased expression of PRB, mPRα and mPRβ may enhance progesterone responsiveness in the MBH on pro-oestrous afternoon and contribute to genomic and/or nongenomic mechanisms underlying the progesterone action with respect to regulating pituitary hormone secretion or other reproductive processes occurring during the pre-ovulatory period.
